# Postural Changes During Exteroceptive Thin Plantar Stimulation: The Effect of Prolonged Use and Different Plantar Localizations

**DOI:** 10.3389/fnsys.2019.00049

**Published:** 2019-09-13

**Authors:** Marco Tramontano, Jacopo Piermaria, Giovanni Morone, Alice Reali, Martin Vergara, Federica Tamburella

**Affiliations:** ^1^Fondazione Santa Lucia IRCCS, Rome, Italy; ^2^Sensor Medica, Guidonia Montecelio, Rome, Italy

**Keywords:** posture, balance, exteroception, plantar, foot

## Abstract

Somatosensory information arising from the foot has an important role in posture as well as visual and vestibular cues. Our hypothesis is that the effects of prolonged stimulation are greater than those of short stimulation and that varying the plantar location can affect postural control. Forty healthy participants were recruited and randomly assigned to four different plantar location groups: Lateral Insert (LI), Medial Insert (MI), Disharmonious Insert (DI), and Central Insert (CI). An instrumental assessment was performed before the plantar stimulation (T0), immediately after the positioning of the inserts (T1), and after 7 days of daily stimulation (T7). A follow-up was performed 15 days after (T15). The following stabilometric parameters were considered for both open eyes (OE) and closed eyes (CE) conditions: length of the sway (L) of the Center of Pressure (CoP); CoP maximum movements in the medio-lateral (X), and antero-posterior directions (Y). Comparing the effects of different plantar insert locations, the MI and CI groups were significantly different in the follow-up measures at T15, specifically for closed eyes measures. When we compared measures across time within each location group, CI group increased measures of X and Y data at T7 compared to other assessment times (T0, T1, and T15). In both MI and LI groups, L was significantly reduced, and X significantly increased at the T7 assessment compared to the T0, T1, and T15 assessments. The prolonged use of exteroceptive plantar stimulation and the location of plantar inserts may have a role to reshape postural control.

## Introduction

Multiple sensory information is involved in the organization of human balance control. In addition to visual and vestibular cues, somatosensory information arising from the foot has an important role in posture (Maurer et al., 2001). This information is integrated by the central nervous system into a continuous sensorial re-weighting, which ensures postural control in both static and dynamic conditions (Bruijn and van Dieën, 2018). The weighting of the sensory inputs likely depends on environmental conditions and it changes according to the motor task being performed by the subject (Perry et al., 2000; Tramontano et al., 2016; Bonnì et al., 2018).

Several studies were carried out to determine the role of plantar receptors in postural control (Bent et al., 2004, 2005; Deshpande and Patla, 2005; O’Connor and Kuo, 2009; McAndrew et al., 2010; Anson et al., 2014). The exteroceptive afferents of the foot sole have an important role in maintaining balance. It is now known that exteroceptive afferents are elaborated by the central nervous system, and integrated with other stimuli, to maintain an erect posture (Kavounoudias et al., 2001). It was found that varying the pressure under the supporting points of the soles modified postural responses to sudden toes-up rotation (Wu and Chiang, 1997). Furthermore, high-frequency vibration of the plantar soles was shown to produce postural reactions (Maurer et al., 2001) and thin plantar inserts (<4 mm high) could induce plantar neuromuscular responses and variations in plantar pressure distribution (Forth and Layne, 2007, 2008; Aminian et al., 2013). Unilateral medial arch support stimulation (3 mm high) was found to induce the perception that the body’s center of mass is shifting toward the stimulated foot (Janin and Dupui, 2009). This response may involve compensatory muscle activation strategies to adjust posture. A recent study (Foisy et al., 2015) analyzed the effects of medial and lateral arch support stimulation with exteroceptive plantar inserts on postural control in standing and on vergence eye movement and found inter-individual variability. Results showed that the central nervous system uses the podal signal for both postural and vergence control through specific mechanisms. Furthermore, movements of ankle inversion and eversion can be modulated by activating muscles through plantar stimulation. In particular, stimulation of the medial arch of the sole of the foot promotes inversion through the activity of the tibialis anterior muscle and triceps surae muscle, while the stimulation of the lateral arch favors eversion through the activity of the long peroneal (Sonnenborg et al., 2000; Stacoff et al., 2007). Several studies (Erkelens et al., 1989; Han and Lennerstrand, 1995; Lennerstrand et al., 1996; Han and Lennerstrand, 1998; Popov et al., 1999) suggested that neck, torso and foot proprioception can affect eye movements by going through a “proprioceptive chain” (Roll and Roll, 1987, 1988). These results could be useful for clinicians when they adopt foot orthoses to address patients’ postural anomalies. Subjects may alternate weight distribution between limbs (Blaszczyk et al., 2000; Haddad et al., 2011), but we are not aware of any evidence suggesting subjects should activate consistently muscles in either leg (Dos Anjos et al., 2018). Indeed, plantar muscles in both limbs can be activated asymmetrically during standing and walking (Liang et al., 2016). Asymmetric plantar pressure distribution between limbs can be found also in healthy subjects (de Paula Lima et al., 2018), but no studies were carried out to investigate the effects of asymmetric plantar stimulation. The effects of immediate plantar stimulation on postural control have been shown (Christovão et al., 2013), but no studies thus far have assessed the potential effect of prolonged stimulation. Our hypothesis is that the effects of prolonged stimulation are greater than those of short stimulation and that other plantar localizations could affect postural control.

Thus, the primary aim of the present study was to investigate the effects of prolonged exteroceptive plantar stimulation on postural control; the secondary aim was to determine whether different plantar stimulations might differently affect postural control in healthy volunteers.

## Materials and Methods

### Ethics Statement

This randomized-controlled single-blinded study was carried out in accordance with the Declaration of Helsinki and was approved by the Local Ethics Committee of Fondazione Santa Lucia IRCCS with protocol number N CE/PROG.601 approved on April 03, 2017.

### Subjects

This preliminary study was conducted at the Operative Unit of Neurorehabilitation 3 of Fondazione Santa Lucia from May 2017 to December 2018. Participants were recruited via a single email invitation from a database of physiotherapy students at Tor Vergata University in Rome. The invitation explained that participation was voluntary, without incentives for participants and dependent on meeting the inclusion and exclusion criteria. All interested participants received information about the project in an interview. A researcher who was not involved in the intervention sessions assessed the eligibility of participants according to the inclusion and exclusion criteria detailed below.

Fifty healthy participants of both genders were recruited. They were non-smokers, aged between 20 and 40 years. They had not undergone any pharmacological treatment during the previous 4 weeks and had not experienced pain based on a VAS score of less than 3 for at least 6 months before enrollment. Participants who had a history of neurological disease, orthopedic problems, had taken medications that affect the central nervous system and who were involved in other research that could potentially affect outcomes, were excluded.

This sample size complied with the minimum number of participants used in previous studies (Foisy et al., 2015; Foisy and Kapoula, 2016).

### Experimental Design

Four groups of participants who were blinded to the type of stimulation were submitted to four different conditions of plantar stimulation. Epidemiological data are reported in Table 1. Participants were randomly divided into four groups according to the stimuli location: Lateral Insert (LI), Medial Insert (MI), Disharmonious Insert (DI) (Bricot, 2011), and Central Insert (CI) (See flow chart into the Supplementary Material). A researcher who was not involved in the intervention sessions performed the randomization and the subsequent statistical analysis. Block randomization was performed according to a computer-generated randomization list using a block size. Allocation concealment was ensured using opaque envelopes. The person responsible for the randomization process deposited the list in secure web-based storage.

**TABLE 1:** Epidemiographic features of enrolled subjects randomized into LI, MI, DI, and CI groups.

**Group**	**N Subjects**	**Mean age (DS)**	**Height/cm**	**Weight/kg**	**Gender**
Lateral Insert (LI)	10	23.60 ± 2.63	174.70 ± 6.75	67.40 ± 10.62	4 F
Medial Insert (MI)	10	23.10 ± 2.60	172.30 ± 8.53	71.00 ± 16.37	6 F
Disharmonious Insert (DI)	10	23.20 ± 1.55	173.50 ± 6.59	66.90 ± 10.56	5 F
Central Insert (CI)	10	24.90 ± 3.70	175.70 ± 8.45	68.00 ± 8.03	3 F

**FIGURE 1:**
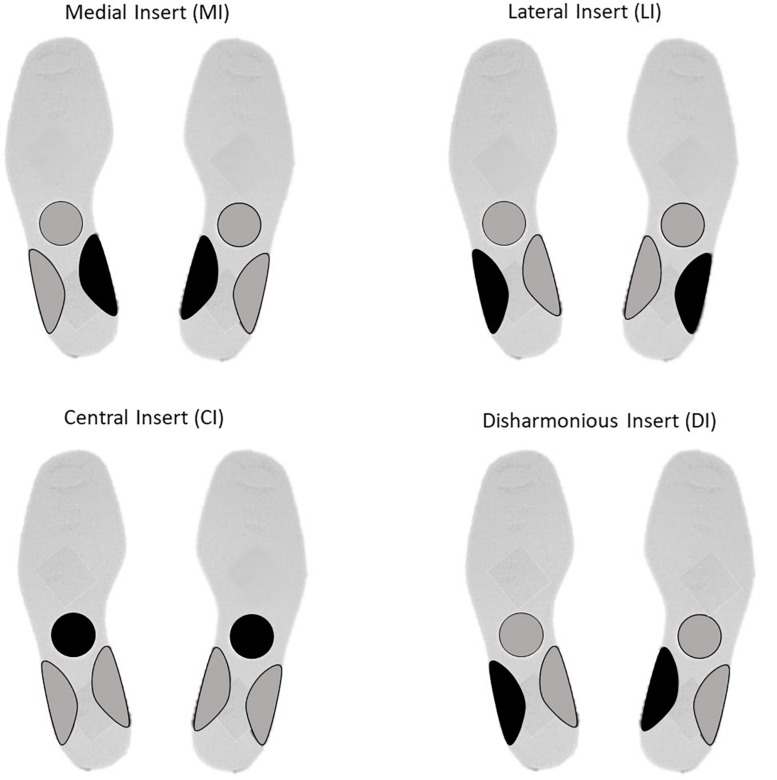
Insert localization for Medial Insert (MI), Lateral Insert (LI), Central Insert (CI), and Disharmonious Insert (DI).

For LI and MI, respectively, a bilateral 3 mm high lateral and medial arch support was adopted (Janin and Dupui, 2009). For the DI group, a 3 mm high arch support was placed under the left medial arch support and a 3 mm high arch was placed under (Bricot, 2011) a right lateral arch support. For the CI group, a round 3 mm high stimulation was placed under the transversal tarsal joint (Chopart’s joint). All plantar inserts are static and were made of rigid polyester resin, with a shore rating of 60A and a density of 250 kg/m3 (Figure 1).

To place the plantar inserts properly under the subjects’ feet, one researcher was specially trained by a physiotherapist with more than 10 years of experience in gait and postural assessment. The physiotherapist was always the same across experimental sessions. The training was about 4 h/day for 2 weeks. To ensure the right placement between T1 and T7, tailored exteroceptive insoles were made for each participant.

Instrumental assessment was performed before the plantar stimulation (T0), immediately after the insert of plantar insoles (T1) and after 7 days of prolonged stimulation (T7). A follow-up was performed 15 days after T1 (T15).

#### Postural Instrumental Assessment

A FreeMed© BASE model baropodometric and stabilometric platform, a product of Sensor Medica, Rome, Italy, was used for measurements. The platform surface was 555 × 420 mm, with an active surface of 400 × 400 mm and 8 mm thickness. The reliability of this baropodometric platform was shown in previous studies (Romero-Franco et al., 2013). Calculations of Center of Pressure (CoP) movements were performed with FreeStep© Standard 3.0 software (a product of Sensor Medica, Rome, Italy). The sensors, coated with 24 K gold, guarantee repeatability and reliability of the instrument (a product of Sensor Medica, Rome, Italy). To eliminate noise, all assessments were performed inside a soundproof booth, like the one used for the audiometric assessment. Participants repeated the static standing measures with open eyes (OE) during the first assessment and with closed eyes (CE) during the second assessment. The analysis was repeated three times for each condition (OE and CE); each one lasted 51.2 s, as detailed previously in the literature (Tamburella et al., 2014).

The following stabilometric parameters were considered for both the OE and the CE conditions: length of the sway (mm) of the CoP (L); CoP maximum movements in the medio-lateral (X), and antero-posterior directions (Y) (Barbero et al., 2012). L was considered the primary outcome, in line with Tamburella et al. (Tamburella et al., 2014), who suggested considering L, related to the duration of the experiment, as the most reliable stabilometric parameter.

### Statistical Analysis

Statistical assessment between four groups was performed on epidemiographic data (age, gender, height, and weight) with one-way Analysis of Variance (ANOVA). For each participant and for each condition three evaluations were made: statistical analysis was performed using the data of 40 subjects who completed all evaluations (240 assessment in total, 6 for each subject). One-way ANOVA was performed to compare differences between groups at T0, T1, T7, and T15. Furthermore, for each group (LI, MI, DI, and CI) stabilometric data collected at different time steps (T0, T1, T7, and T15) were compared with ANOVA. When ANOVA results reached significance, a Bonferroni *post hoc* test was performed. Statistical significance was considered at *p* < 0.05. All statistical tests were performed using the Statistical Package for the Social Sciences Software (SPSS), version 12.0 (Chicago, IL, United States).

## Results

Before statistical comparisons were made, a Kolmogorov–Smirnov test was performed to evaluate the distribution of the data. We screened fifty healthy subjects, and according to inclusion/exclusion criteria, 40 subjects were enrolled (see flow chart in the Supplementary Material). No statistical differences between groups were pointed out with ANOVA assessment in terms of age, gender, height, and weight.

**FIGURE 2:**
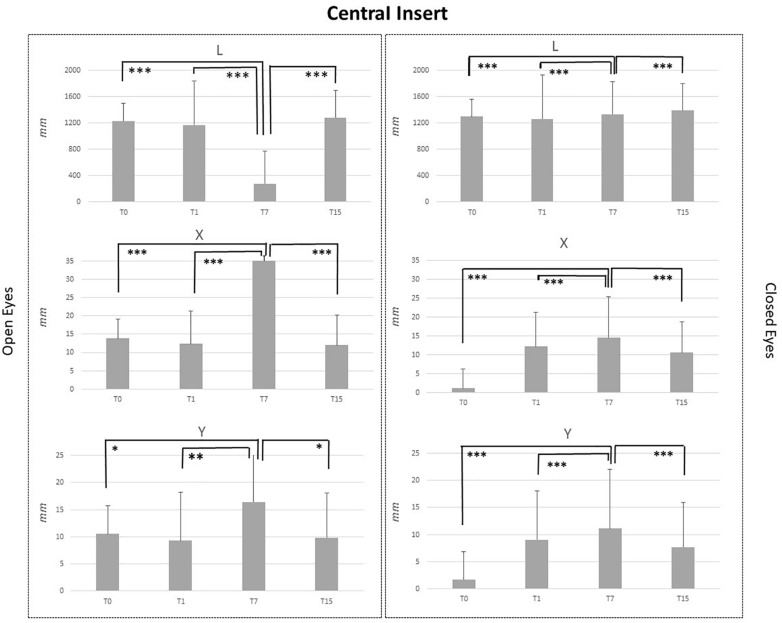
L, X, and Y data assessed in both conditions, open eyes and closed eyes, with CI at T0, T1, T7, and T15. Comparison between T0, T1, T7, and T15 is reported above bars. ^∗^*p* < 0.05, ^∗∗^*p* < 0.005, ^∗∗∗^*p* < 0.001.

**FIGURE 3:**
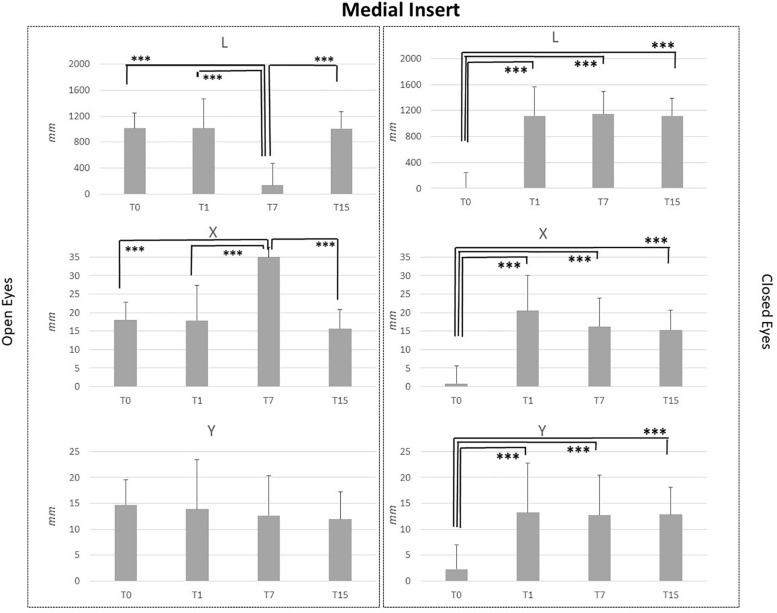
L, X, and Y data assessed in both conditions, open eyes and closed eyes, with MI at T0, T1, T7, and T15. Comparison between T0, T1, T7, and T15 is reported above bars. ^∗^*p* < 0.05, ^∗∗^*p* < 0.005, ^∗∗∗^*p* < 0.001.

**FIGURE 4:**
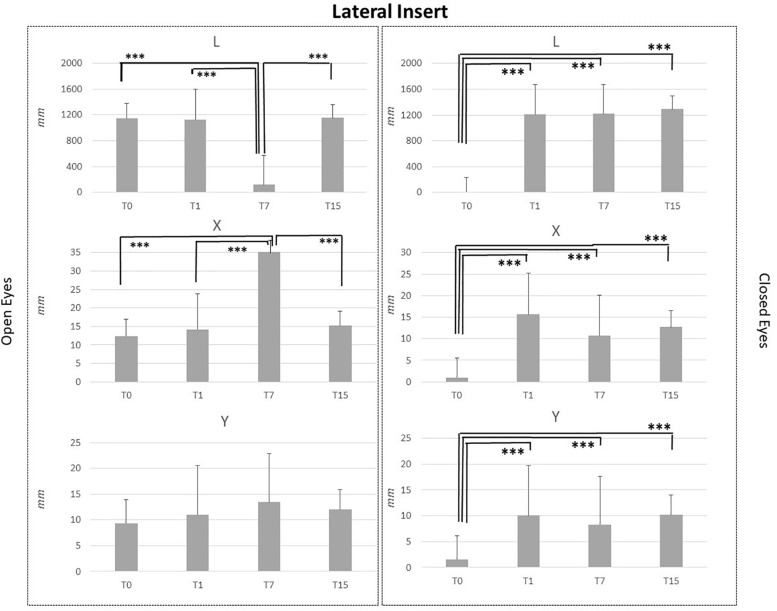
L, X, and Y data assessed in both conditions, open eyes and closed eyes, with LI at T0, T1, T7, and T15. Comparison between T0, T1, T7, and T15 is reported above bars. ^∗^*p* < 0.05, ^∗∗^*p* < 0.005, ^∗∗∗^*p* < 0.001.

**FIGURE 5:**
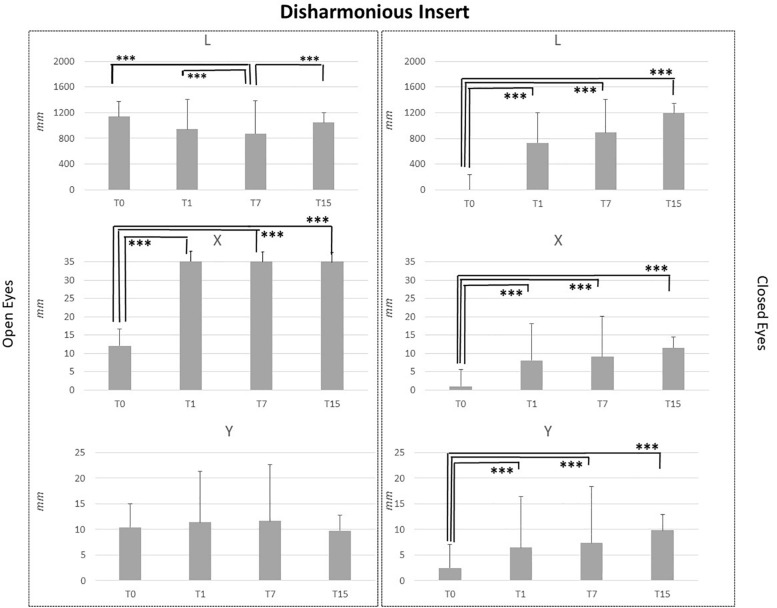
L, X, and Y data assessed in both conditions, open eyes and closed eyes, with DI at T0, T1, T7, and T15. Comparison between T0, T1, T7, and T15 is reported above bars. ^∗^*p* < 0.05, ^∗∗^*p* < 0.005, ^∗∗∗^*p* < 0.001.

Baseline comparison (T0) between groups showed no statistical differences as well as for the T1 and T7 comparisons. It is intriguing that differences between groups obtained at T15 CI and MI groups differed for the following variables: L_OE (*p* = 0.006), L_CE (*p* = 0.03), and Y_CE (*p* = 0.004). Statistical differences for each group at T0, T1, T7, and T15 are reported in Figures 2–5.

CI application generated significant effects for both OE and CE conditions. Concerning the OE condition, T7 vs. T0, T7 vs. T1 and T7 vs. T15 comparisons pointed out a significant reduction of L values (*p* < 0.001) and a significant increment of X (*p* < 0.001) and Y values (*p* < 0.05: Y_T7 vs. Y_T0 and Y_T15; *p* < 0.005; Y_T7 vs. YT1). For the CE condition, results were similar (Figure 2).

MI and LI application in healthy subjects affected in a similar way stabilometric data. MI application did not disturb Y parameter in the OE condition, while some effects were noted for L and X data. Comparison between T7 vs. T0, T7 vs. T1 and T7 vs. T15 assessments in OE condition, pointed out a significant reduction of L (*p* < 0.001) and a significant increment of X data (*p* < 0.001). Concerning the CE condition for all analyzed parameters, significant differences were highlighted for L, X, and Y data in the comparisons between T0 vs. T1, T0 vs. T7 and T0 vs. T15 data. L_T0, X_T0, and Y_T0 data were significantly lower than L, X, and Y data recorded at T1, T7 and T15 (*p* < 0.001). No differences were present between T1, T7, and T15 assessments for L, X, and Y data in the CE condition.

LI effects data suggested that subjects affected stabilometric data in a similar way to MI ones, as reported in Figure 4. Stimuli applications did not affect Y parameter in the OE condition, even T7 vs. T0, T7 vs. T1 and T7 vs. T15 assessments comparisons pointed out a significant L reduction (*p* < 0.001) and a significant X increment (*p* < 0.001). Concerning the CE condition significant differences were pointed out for L, X, and Y data in the T0 vs. T1, T0 vs. T7 and T0 vs. T15 comparisons. L, X, and Y data at T0 were significantly lower than L, X, and Y data at T1, T7, and T15 (*p* < 0.001). No differences were present between T1, T7, and T15 assessments for L, X, and Y data in the CE condition. See Figure 4 for LI data.

DI application did not affect Y data in the OE condition, but significant differences emerged for L_OE at T7 vs. T0, T7 vs. T1 and T7 vs. T15 assessment comparisons (*p* < 0.001) and for X_OE at T0 vs. T1, T0 vs. T7 and T0 vs. T15 assessment comparisons. Regarding CE condition, the same pattern of MI and LI was recorded. For all analyzed parameters, L, X, and Y, T0 data were significantly lower than those recorded at T1, T7, and T15 (*p* < 0.001). No differences were present between T1, T7, and T15 assessments for L, X, and Y data in the CE condition as reported in Figure 5.

For specific details about L, X, and Y data for CI, MI, LI, and DI effects see table in the section “Supplementary Material.”

## Discussion

The aim of this study was to investigate the effects of different types of prolonged plantar stimulation on postural control in healthy young volunteers. The results of our study indicate that exteroceptive plantar afferents can be stimulated by plantar inserts of 3 mm high, confirming previously reported data (Kavounoudias et al., 2001; Foisy and Kapoula, 2016). In our study, the greatest effects were produced when the stimulus was maintained over time; this was found primarily in the detection at T7 in the CI and MI groups.

These findings highlight the importance of not only medial and lateral inserts in influencing the postural control but also of central and disharmonious inserts. Our results could support the hypothesis that there is a relationship between the effectiveness of the plantar inserts and the duration they are applied.

It is also interesting that the significant differences between groups were obtained only in relation to T15 in the MI and CI groups, in particular in the L_OE (*p* = 0.006), L_CE (*p* = 0.03), and Y_CE (*p* = 0.004) parameters, supporting the hypothesis of an inter-individual variability regarding how plantar exteroceptive efficiency can modulate postural control. Several authors have shown that the application under the sole of the foot produces postural reactions (Kavounoudias et al., 1998) and that likewise a thickness of less than 4 mm can induce neuromuscular responses (Forth and Layne, 2007, 2008).

Another study showed that thin plantar stimulation is associated with a variation in the distribution of plantar pressure on the ground (Aminian et al., 2013). This finding, which was achieved with minimal thickness, can first be explained by the enhancement of the cutaneous afferents to the postural system due to the 3-mm difference of height created by the plantar inserts and detected by the numerous mechanoreceptors of the plantar skin (Kennedy and Inglis, 2002). Furthermore, the addition of a subliminal stimulus in a non-linear system can improve its sensitivity (Fallon and Morgan, 2005) suggesting that plantar stimulation is a precious clue that central nervous system uses to assess the position of the body.

In other words, it is more likely that a calibrated extrinsic stimulus (which is added experimentally) will more easily correspond to the optimal threshold of the receptors. This is associated with a perceptual amplification and a more efficient motor response (Collins et al., 1997). Moreover, the plantar stimulation can influence modulation of the inversion and eversion movements at the ankle level depending on whether the proprioceptive stimulus is inserted under the medial or lateral arch of the foot. In particular, the stimulation of the medial arch of the sole of the foot favors inversion through the activation of the tibial anterior muscle and triceps surae muscle whereas the stimulation of the lateral arch favors eversion especially through the activity of the long peroneal muscle (Sonnenborg et al., 2000). The proprioceptive information should not be considered as a system of isolated afferents but in relation to other systems such as vision and oculomotor afferents. In fact, ocular vergence movements and podalic information influence each other in order to guarantee postural stability (Foisy et al., 2015). In normal conditions, there is a real visual-podalic synergy in which both signals are easily used and equally processed by the central nervous system, producing efficient postural control (Foisy and Kapoula, 2017). In fact, the same authors show that in the event that one of the two signals is altered, the other cannot be used optimally. These authors also compared the responses of two groups of people: the first group was characterized by a condition of normality in which the individuals’ visual, podalic signals and oculomotor system were not altered; the second group was characterized by individuals who had a podalic dysfunction.

The latter condition causes an increase in pressure below some specific areas of the foot (especially at the level of the metatarsal heads), with a consequent increase in the frequency of discharge of podalic receptors. These results confirm that plantar inserts have the capacity to modulate podal feedback. In fact, they lead us to consider them as they vary not only in the application procedure and area but also in the type of stimulus in relation to the dysfunction of the foot.

The variability of our results, which were obtained by comparing the different sites of exteroceptive inserts application and different stabilometric parameters, could be related to how much subjects use plantar afferents. In fact, in the healthy young subjects, there are inter-individual sensorimotor and perceptive differences. Some of them are mainly based on visual and vestibular inputs, postural control and spatial perception (Crémieux and Mesure, 1994; Lacour et al., 1997; Ehrenfried et al., 2003; Isableu et al., 2010, 2011). In support of this hypothesis, Foisy and Kapoula (2016) recently demonstrated the existence of inter-individual variability regarding how plantar exteroceptive efficiency modulates postural and oculomotor control, which is explained by the subjects’ degree of plantar reliance. According to the Authors, these differences could be related to the inability to correctly use the proprioceptive afferents from the feet, a clear expression of the presence of a “dysfunction” of the foot that would cause an alteration (in both excess and defect) of the signal. This variability is reduced at T7 where all the study groups showed a significant change in stabilometric parameters, thus supporting the hypothesis of a correlation between the duration of the application and the effectiveness of plantar inserts.

The different effects due to stimulation positioning on the most reliable stabilometric parameter, L, are intriguing. With CE, CI allowed a significant increase in L data at T7, whereas LI and MI allowed a significant increase of L already at T1 as well as DI. Concerning the OE condition, L was significantly reduced at T7 in comparison to T0 for CI, LI, MI, and slightly also for DI. These results might suggest the presence of different sensory influences due to different stimulations. It is possible that the effect of plantar stimulation occurred on the first day with CE but took longer to be revealed with OE.

Significant differences for measures under open or closed eye conditions were observed. This variability could be related to a decrease in visual and oculomotor afferents that increasing the use of plantar cues facilitate a somatosensory integration. The visual-podal synergy/asynergy could justify different behavior under open or closed eyes condition also in healthy subjects (Foisy and Kapoula, 2017). These findings may have clinical implications because subjects with plantar dysfunction may be more unstable and have trouble to integrate both their plantar and visual afferents.

Further studies are needed to clarify this point, but it is possible that the removal of eye afferents would reveal stimulation effects immediately. This is in line with results of a previous study in which it was noted that the CE condition assessment is more valid for detecting changes in balance control (Tamburella et al., 2014).

These results suggest the need to categorize various aspects. First, we need to identify the inter-individual variability concerning the use of podalic afferents and to objectify these clinical entities and their neurophysiological characteristics in order to obtain greater homogeneity in the study groups. It would be interesting to verify their effects on dysfunctions. Therefore, it would be interesting to study healthy subjects without dysfunctions using the same experimental method, which alters the afferents from the feet, to analyze the strength of the link between the application methods or procedures of the stimulators and the variation of the stabilometric parameters. Subsequently, it would be useful to recruit subjects with podalic dysfunctions to discover the potential clinical efficacy of plantar exteroceptive stimulation.

A recent review (Viseux et al., 2019) reports that there is a relation between balance improvement and the facilitation of sensory feedback related to the activation of the plantar cutaneous mechanoreceptors. From a clinical point of view, the application of thin plantar inserts may have therapeutic benefits in relation to balance disorders, or to improve specific types of chronic pain.

### Study Limitation

Even if results are intriguing some limitations must be considered. We selected the stabilometric evaluation as an instrumental assessment for outcome measures study because this is the most common instrument used in the clinical practice. This study did not consider the individual subject differences with the related variability in the measurements, but within-subject variability may have been reduced from the three measures per subject per condition.

Besides, no specific questionnaires devoted to assessing the personal perception of the patient in terms of possible discomfort or feeling due to the different inserts localization were administered. Furthermore, the effect of the tailored exteroceptive insoles without the thin plantar inserts is not evaluated and this could be a bias. Future studies that address sources of variability may include assessments of individual subject perception and stabilometric parameters while wearing the tailored insoles with and without the thin plantar inserts.

## Conclusion

This study highlights new insights in the rehabilitation context suggesting that the application of thin plantar inserts may have different effects according to the location and the duration of stimulation. Furthermore, these findings confirm that stabilometric assessment is an easy way to assess postural stability of subjects. The prolonged use of exteroceptive plantar stimulation and the location of plantar inserts may have a role to reshape postural control.

## Data Availability

The data that support the findings of this study are available from the corresponding author MT, upon reasonable request.

## Ethics Statement

The studies involving human participants were reviewed and approved by the Local Ethics Committee of Fondazione Santa Lucia IRCCS with protocol number N CE/PROG.601 approved on April 03, 2017. The patients/participants provided their written informed consent to participate in this study.

## Author Contributions

MT, FT, and JP organized the database. MT and FT performed the statistical analysis. MT and GM wrote the first draft of the manuscript. JP, GM, AR, MV, and FT wrote the sections of the manuscript. All authors contributed to the conception and design of the study, revision of the manuscript, and reading and approving the submitted version.

## Conflict of Interest Statement

The authors declare that the research was conducted in the absence of any commercial or financial relationships that could be construed as a potential conflict of interest.
