# Supplementary figures and images for: Postural Changes During Exteroceptive Thin Plantar Stimulation: The Effect of Prolonged Use and Different Plantar Localizations
Source: Front Syst Neurosci. 2019 Sep 13;13:49. doi: 10.3389/fnsys.2019.00049 (PMC6753192; doi:10.3389/fnsys.2019.00049)

## Supplementary Material

### Flow Chart

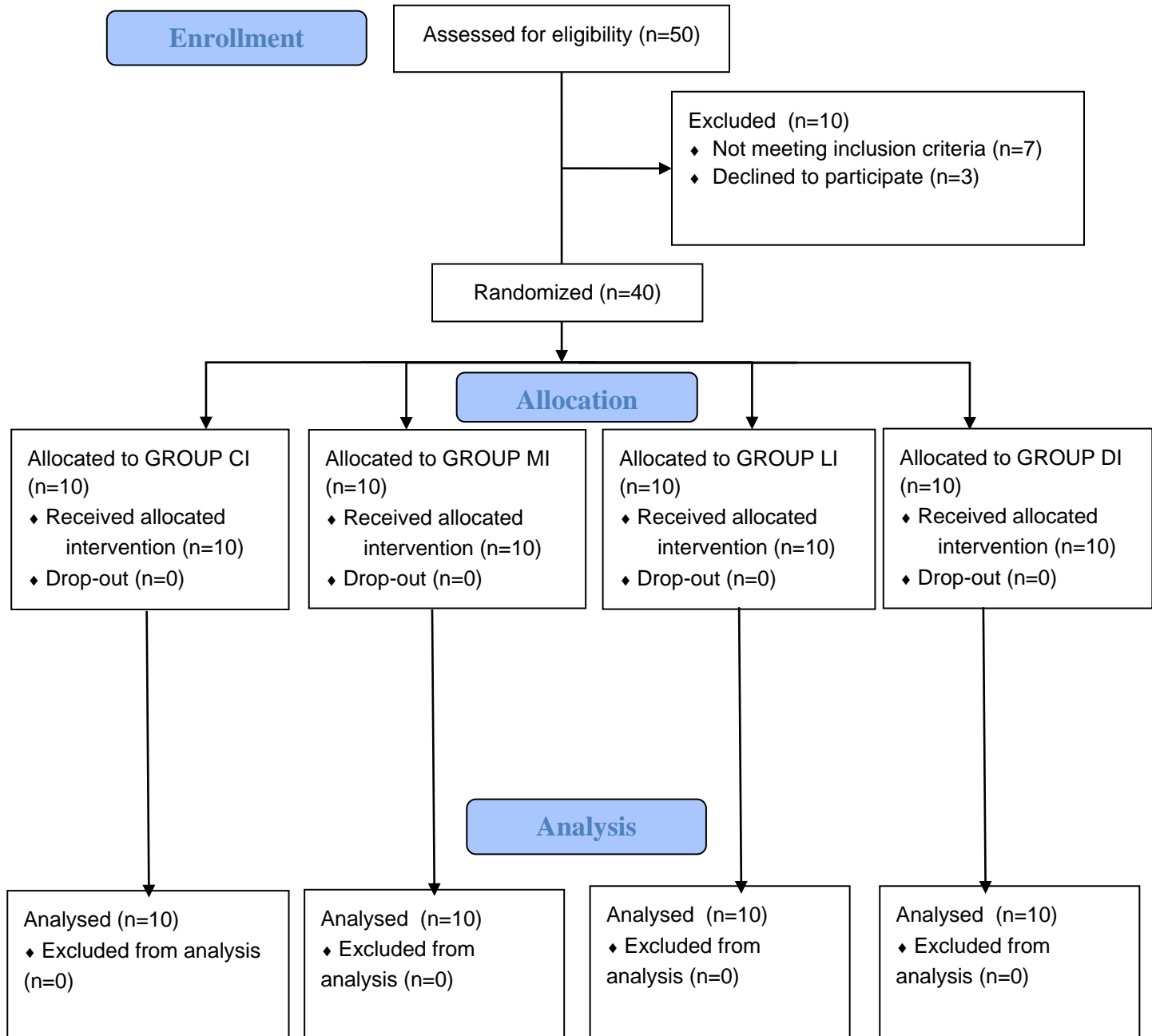

Supplement: Supplementary file 1 [file Data_Sheet_1.pdf]
