# Supplementary material for: Postural Changes During Exteroceptive Thin Plantar Stimulation: The Effect of Prolonged Use and Different Plantar Localizations
Source: Front Syst Neurosci. 2019 Sep 13;13:49. doi: 10.3389/fnsys.2019.00049 (PMC6753192; doi:10.3389/fnsys.2019.00049)
Supplement: Supplementary file 2 [file Data_Sheet_2.pdf]

Supplementary Material

Stabilometric parameters for each condition

	OE			CE		
	L	X	Y	L	X	Y
**CI**						
**T0**	1230±264.8	13.9±8.61	10.5±5.79	1297±247.2	1.1±7.54	1.7±6.15
**T1**	1170.5±668.09	12.4±10.09	9.2±14.25	1260.7±297.05	12.2±8.2	9.1±9.2
**T7**	279.8±493.7	34.9± 7.7	16.4±15.4	1334.4±384.6	14.5±12.2	11.1±14
**T15**	1279±410.8	12±17.3	9.8±13.1	1387.7±674	10.5±5.3	7.7±8.1
**MI**						
**T0**	1011.5±239.4	18.0±7.1	14.7±5.8	2.4±331.2	0.9±5.6	2.2±5.3
**T1**	1012.4±452.3	17.9±12.7	13.9±8.7	1117.1±369.2	20.5±11.5	13.2±9.3
**T7**	133.1±340.7	35.7±2.3	12.6±7.2	1150.9±282.7	16.2±9.2	12.8±7.9
**T15**	1006.2±268.1	15.6±6.3	11.9±5.3	1116.6±225.1	15.4±6.1	12.8±5.4
**LI**						
**T0**	1143.8±232.9	12.4±6.3	9.3±5.6	2.4±321.1	1.1±6.1	1.5±7.2
**T1**	1129.1±465.9	14.2±9.4	11.0±9.9	1208.2±445.6	15.6±11.5	10.1±8.9
**T7**	118.6±453.6	36.3±4.2	13.4±10.5	1220.2±414.3	10.7±10.2	8.3±12.4
**T15**	1155.7±199.1	15.2±5.2	12.2±5.4	1292.7±180.6	12.7±4.1	10.2±4.3
**DI**						
**T0**	1137.5±235.2	12.1±9.2	10.4±8.2	2.6±328.4	1.0±6.9	2.5±6
**T1**	939.9±467	36.9±4.8	11.4±10.4	732.7±521.1	8.1±7.9	6.4±10.7
**T7**	873.1±513.4	35.4±5.4	11.6±14.7	893.7±571.8	9.1±8.5	7.3±15.8
**T15**	1048.7±153.3	36.6±7.2	9.7±4.8	1193.9±160.6	11.5±4.2	9.8±4.7

Mean±standard deviation of stabilometric parameters for each condition;

OE=Open Eyes; CE= Closed Eyes; CI= Central Insert; MI= Medial Insert; LI= Lateral Insert; DI= Disharmonious Insert

L= length (mm) of the sway of the Center of Pressure (CoP) ; X= mean position of CoP alongthe medio-lateral plane; Y= mean position of CoP along the antero-posterior plane.
